# Professional Education

**Published:** 1879-06

**Authors:** 


### Editorial.

### PROFESSIONAL EDUCATION.

The following, from "The Physician and Surgeon," contains matter for thought and reflection for every one.

The criticisms and suggestions here presented, in reference to the education and licensing the general physician, applies with equal force to those engaged in any specialty of the healing art; and to the dentist quite as much as to any other. The same difficulty and embarrassment attach to nearly all medical colleges in this country.

In nearly all medical and dental colleges there is sharp and overweaning competition for students; the common results of this competition is the letting down of requirements and qualifications, and the acceptance of students totally unqualified as to preliminary attainments for undertaking and prosecuting medical studies.

The suggestion that the colleges should be limited to teaching, and that the licensing for practice should be deferred to other hands, is one worthy of careful consideration.

That the system of medical education and licensing for practice, needs some radical change, is very apparent from the fact that not more than one-half who are in the practice of medicine or dentistry are in any proper sense prepared for the work in which they are engaged. So far as the dental profession is concerned the proportion of incompetents is much larger than that just given.

Who is responsible for this state of things?

First, the profession; and second, the colleges.

What shall be the remedy?

"A FEW FACTS.

"In France, with a population of forty million, there are only five bodies that can confer the right to practice medicine, *i. e.*, the faculties of medicine of Paris, Montpelier,

Nancy, Lyons and Lille. In Germany not a college, we believe, has this right. The colleges give the degree of doctor of medicine; but before one is permitted to practice in that country he must have passed an examination before the State Board, 'Staats Examen.' The requirements to enter a medical college in Great Britian, to say nothing of those demanded at the close of the medical course, are well known to every reading physician. How many bodies there are in the United States that can confer the right to practice medicine would be difficult to say. In New York State, with a population of four million seven hundred thousand, there are thirteen medical colleges and *many* country societies that can grant this right to almost any person. 'There are but few people who are aware that any five persons of a suitable age may associate together under the general act for incorporating charitable, benevolent and scientific societies, and establish a medical college with teachers possessing no adequate qualifications, but whose diploma will be as valid here as that of our best and oldest colleges.' By this slack method of dealing out medical degrees, the vast disproportion in this country between medical practitioners and the population is becoming somewhat alarming; at least, so far as the profession is concerned, as is shown by the following figures: In Italy there is one physician to three thousand five hundred people; in Norway, one to three thousand four hundred and eighty; in Austria, one to two thousand five hundred; in Belgium, one to two thousand and forty-eight; in France, one to one thousand eight hundred and fourteen; in Great Britian, one to one thousand six hundred and seventy-two; in Canada, one to one thousand two hundred; in the United States, one to six hundred. This is, however, a secondary matter. The greater harm can not come to the physician, but to the public who have to place themselves in the hands of the doctor, without means of knowing whether he is good, bad or indifferent, until, perhaps, it is too late. This is well understood by physicians, the honest portion of whom is trying every way in its power to enlighten the public, while another class,

*out of fear*, is doing all in its power to keep the people in ignorance of the facts. We heartily agree with the one who has suggested the following to ameliorate this state of things: '(1) The licensing power should be taken away from the medical societies. (2) The professors in the medical colleges should not be dependent for their emoluments upon the number of students they can get and graduate. (3) Before a student is allowed to matriculate, he should show that he has a good English education and some knowledge of the liberal sciences and Latin. (4) The length of the course of study should be increased.' The same person says further: 'The greatest reform, however, and one which must come in time will be to give the licensing power to State Boards of Examiners, making the colleges only the vehicles of instruction. Harvard University, the University of Pennsylvania and the medical college of Syracuse have taken vast strides in making their curiculums more thorough, and inaugurating reforms which I can not here touch upon. Among the above named universities should have appeared the name of the University of Michigan, which institution has lengthened its course, requires an examination of its students before matriculation, whose professors do not depend for support upon the number of students in attendance, and whose students are examined by a "State Board of Examiners." When each State has formed a law, and enforces it, that no person shall practice medicine, unless he or she has received a degree from an institution adopting and adhering to the above or more thorough propositions, the favorite note of the duck will not be so familiar to the ears of the American people, and prussic acid will not be 'administered in whooping cough to a child two years old in doses from four to six drops.' R. W. C."

